# Association between circulating levels of C-reactive protein and positive and negative symptoms of psychosis in adolescents in a general population birth cohort

**DOI:** 10.1016/j.jpsychires.2020.11.028

**Published:** 2021-11

**Authors:** Golam M. Khandaker, Jan Stochl, Stanley Zammit, Glyn Lewis, Robert Dantzer, Peter B. Jones

**Affiliations:** a Department of Psychiatry, University of Cambridge, Herchel Smith Building, Forvie Site, Cambridge Biomedical Campus, Cambridge, CB2 0SZ, United Kingdom; b Cambridgeshire and Peterborough NHS Foundation Trust, Elizabeth House, Fulbourn Hospital, Fulbourn, Cambridge, CB21 5EF, United Kingdom; c Department of Kinanthropology, Charles University, José Martího 31, Prague, 165 52, Czech Republic; d Centre for Academic Mental Health, Department of Population Health Sciences, Bristol Medical School, University of Bristol, Oakfield House, Oakfield Grove, Bristol, BS8 2BN, United Kingdom; e Institute of Psychological Medicine and Clinical Neurosciences, Cardiff University, Cardiff, CF10 3AT, United Kingdom; f Division of Psychiatry, University College London, Maple House, 149 Tottenham Court Road, London, W1T 7NF, United Kingdom; g Department for Symptom Research, University of Texas MD Anderson Cancer Center, Laboratory of Neuroimmunology, Unit 1055, Office: Z8.5042, 6565 MD Anderson Boulevard, Houston, TX, 77030, USA

**Keywords:** ALSPAC, Inflammation, C-reactive protein, Psychotic symptoms, Negative symptoms, Cohort study, IL-6, interleukin 6, CRP, C-reactive protein, PTSD, post-traumatic stress disorder, ALSPAC, Avon Longitudinal Study of Parents and Children, SD, Standard Deviation, BMI, body mass index, SMFQ, Short Mood and Feelings Questionnaire, EFA, Exploratory Factor Analysis, CFI, Comparative Fit Index, TLI, Tucker-Lewis Index, RMSEA, Root Mean Square Error of Approximation, OR, Odds Ratio, CI, Confidence Interval, IQR, Interquartile Range, IDO, indoleamine 2,3 dioxygenase, NMDAR, N-methyl D-Aspartate Receptor, CNS, Central Nervous System, CSF, Cerebrospinal Fuild, RCT, Randomised Controlled Trial

## Abstract

**Background:**

Schizophrenia is associated with elevated levels of circulating C-reactive protein (CRP) and other inflammatory markers, but it is unclear whether these associations extend to psychotic symptoms occurring in adolescence in the general population. A symptom-based approach may provide important clues for apparent trans-diagnostic effect of inflammation, which is also associated with depression and other psychiatric disorders.

**Methods:**

Based on data from 2421 participants from the Avon Longitudinal Study of Parents and Children birth cohort, we examined associations of serum CRP levels assessed around age 16 with ten positive and ten negative symptoms of psychosis assessed using questionnaires around age 17, using both individual symptoms and symptom dimension scores as outcomes. Regression models were adjusted for sex, body mass index, depressive symptoms, substance use, and other potential confounders.

**Results:**

Most prevalent positive symptoms were paranoid ideation (4.8%), visual (4.3%) and auditory (3.5%) hallucinations. Negative symptoms were more strongly correlated with concurrent depressive symptoms (*r=*0.51; *P* < 0.001) than positive symptoms (*r*_pb_*=*0.19; *P* < 0.001). The associations of CRP with positive and negative symptom dimension scores were similar. At individual symptom level, after adjusting for potential confounders including depressive symptoms, CRP was associated with auditory hallucinations (adjusted OR = 2.22; 95% CI, 1.04–4.76) and anhedonia (adjusted OR = 1.13; 95% CI, 1.02–1.26).

**Conclusions:**

Inflammation is associated with sub-clinical psychotic symptoms in young people in general population. Association of CRP with symptoms commonly shared between mood and psychotic disorders, such as auditory hallucinations and anhedonia, could be one explanation for the apparent trans-diagnostic effect of inflammation.

## Highlights

• We examined psychotic symptoms in adolescents from a population-based birth cohort.
• Common positive symptoms were paranoid ideation, visual and auditory hallucinations.
• Negative symptoms were correlated with depressive and positive symptoms.
• CRP was similarly associated with positive and negative symptom dimension scores.
• CRP was specifically associated with auditory hallucinations and anhedonia.

## 1 Introduction

Low-grade systemic inflammation as reflected by increased concentrations of acute phase proteins, such as C-reactive protein (CRP), and inflammatory cytokines, such as interleukin 6 (IL-6), in peripheral blood has been implicated in pathogenesis of a number of psychiatric disorders including schizophrenia and related psychoses (Khandaker et al., 2015; Miller et al., 2011), depression (Dantzer et al., 2008; Miller et al., 2009), anxiety (Wohleb et al., 2014), post-traumatic stress disorder (Eraly et al., 2014), autism (Brown et al., 2014), Alzheimer's disease and other dementias (Schmidt et al., 2002). However, possible reasons for this apparent trans-diagnostic association of inflammation are unknown. Most human studies are based on categorical diagnosis of illness, but a symptom-based approach may help to elucidate why association between inflammation and psychiatric disorders transcends traditional diagnostic boundaries. It is possible that inflammation contributes to pathogenesis of symptoms that are shared between disorders.

There is a rich literature linking infection and elevated levels of inflammatory markers with the risk of schizophrenia (Benros et al., 2011; Brown and Derkits, 2010; Buka et al., 2001; Dickerson et al., 2003; Khandaker et al., 2012, 2013, 2015; Mortensen et al., 2007; Prasad et al., 2012). Meta-analysis of cross-sectional studies confirm that concentrations of circulating CRP, IL-6 and other inflammatory markers are elevated during an acute psychotic episode (Miller et al., 2011, 2013; Potvin et al., 2008; Upthegrove et al., 2014), which tend to normalize after recovery but continue to be elevated in treatment resistant patients (Goldsmith et al., 2016). Longitudinal studies reporting an association between higher blood IL-6, CRP, erythrocyte sedimentation rate (ESR) in childhood/adolescence and increased risks of psychotic symptoms (Khandaker et al., 2014) or diagnosis of schizophrenia (Kappelmann et al., 2019; Metcalf et al., 2017) subsequently in adulthood indicate that inflammation may play a role in causing psychosis rather than simply being a consequence of illness.

Psychotic disorders consist of diverse symptoms, some of which are shared by other disorders. For instance, auditory hallucinations can occur in psychosis, depression and anxiety disorders (APA, 2013). Anhedonia is both an important negative symptom for psychosis and a core feature of depression (APA, 2013). However, studies examining association between inflammation and specific symptoms of psychosis are limited. Existing studies have reported an association of CRP with negative symptoms (Boozalis et al., 2017), general psychopathology (Fan et al., 2007) and cognitive dysfunction (Dickerson et al., 2007; Johnsen et al., 2016) in patients with psychosis. Psychotic symptoms reported by adolescents and adults in general population thought to exist in a continuum with clinical psychosis (van Os et al., 2009), and are associated with a range of psychotic and non-psychotic disorders cross-sectionally and longitudinally (Kelleher et al., 2012b; McGrath et al., 2016; Poulton et al., 2000; Zammit et al., 2013). However, to our knowledge no population-based studies have examined the association between CRP and positive and negative symptoms of psychosis in a general population-based sample.

We present a longitudinal study of serum CRP concentration assessed around age 16 years and subsequent self-reported positive and negative symptoms of psychosis assessed around age 17 years in the Avon Longitudinal Study of Parents and Children (ALSPAC), a general population-representative birth cohort from the United Kingdom. We hypothesised that elevated CRP levels would be associated with specific psychotic symptoms commonly shared by other disorders. We used individual symptoms and factors scores for positive and negative symptom dimensions as our outcomes. Mood symptoms often co-occur with psychotic symptoms (Stochl et al., 2015) and are associated with elevated inflammatory markers (Howren et al., 2009), as are smoking, alcohol, cannabis and other drug use. We have adjusted for these important confounders, and carried out sensitivity analyses after excluding participants who reported positive symptoms in the context of drugs, physical illness or sleep.

## 2 Methods

### 2.1 Sample

The ALSPAC birth cohort initially recruited 14541 pregnant women resident in study catchment areas in Southwest England who had expected delivery dates between April 1, 1991 and December 31, 1992, resulting in 14062 live births. Additional children and parents were subsequently recruited into the cohort. Since age 7 years, the children attended annual clinical assessments during which they participated in various face-to-face interviews and physical tests. Detailed information about the ALSPAC cohort can be found on the study website (http://www.bristol.ac.uk/alspac), and the sample characteristics and methodology have been described previously (Boyd et al., 2013; Fraser et al., 2013). For information on all available ALSPAC data, see the fully searchable data dictionary (http://www.bris.ac.uk/alspac/researchers/data-access/data-dictionary). The risk set for the current study comprised 3488 participants with data on serum CRP levels around age 16 years. All analysis was based on maximum available sample (see Tables). The analysis for association of CRP with positive and negative symptoms comprised 2421 and 2419 participants, respectively. Ethical approval for the study was obtained from the ALSPAC Ethics and Law Committee and the Local Research Ethics Committees.

### 2.2 Measurement of high sensitivity CRP

Blood samples were collected from participants who gave consent for venepuncture during clinical assessment at average age 15.5 years (SD = 0.35). Participants fasted overnight before attending the clinic if seen in the morning, or at least for 6 h if seen in the afternoon. Blood samples were immediately spun, frozen and stored at −80 °C, which were analysed within 3–9 months of blood sampling with no freeze-thaw cycles in between. High sensitivity CRP (hsCRP) was measured by automated particle-enhanced immunoturbidimetric assay (Roche UK, Welwyn Garden City, UK). No other inflammatory makers were measured. Among all 3488 participants, serum CRP level ranged from 0.07 to 72.55 mg/L; 62 participants (1.77%) had CRP levels ≥10 mg/L. Based on the American Heart Association and the US Centers for Disease Control and Prevention guidelines on the use of hsCRP levels in epidemiological studies (Pearson et al., 2003), hsCRP was coded as a categorical variable: <1 mg/L = “low”; 1–3 mg/L = “medium”; >3 mg/L = “high”. hsCRP was also used as a continuous variable.

### 2.3 Assessment of positive symptoms

Participants completed a questionnaire at average age 16.6 years (SD = 0.23), which included 10 questions about positive symptoms of psychosis asking whether they had experienced these symptoms at least once since 15th birthday (eTable 1). The questionnaire was modelled on the psychotic-like symptoms interview (PLIKSi) which was administered in the same cohort at age 12 years (Zammit et al., 2013). The symptoms elicited can be classed under three major domains: *hallucinations* (auditory; visual); *delusions* (paranoid beliefs to have been followed/spied on; belief someone else has read their thoughts; feeling that they were someone really special or had special powers, i.e., grandiosity; belief to have received messages from television/radio, i.e., ideas of reference; feeling of being under the control of a special power, i.e., passivity); *thought interference* (thought insertion, withdrawal, and broadcasting). For each question, participants were given three options: definitely yes; may be; no, never. Cases were defined as participants reporting to have *definitely* experienced a psychotic symptom. The other two groups (yes, may be and no, never) were merged and used as control. This is because due to lack of cross-questioning, as in an interview, we are less confident whether those reporting “may be” in the questionnaire indeed experienced a positive symptom.

### 2.4 Assessment of negative symptoms

Negative symptoms were assessed using a questionnaire, which included 10 questions based on items from the validated Community Assessment of Psychic Experiences (CAPE) questionnaire (Stefanis et al., 2002), administered at the same time as the questionnaire for positive symptoms. These covered difficulties with interest, motivation, emotional reactivity, pleasure, and sociability. Participants rated each item on a 4-point scale (0 = never; 1 = sometimes; 2 = often; and 3 = always) (eTable 2). We were interested in symptoms that were more frequent, so perhaps clinically relevant. Therefore, we recoded each item into a binary variable by coding ‘often’ and ‘always’ as 1 = symptom present; ‘never’ and ‘sometimes’ as 0 = symptom absent. A total score was constructed by summing ten items (range 0–10).

According to empirical research (Messinger et al., 2011) and the US National Institute of Mental Health expert consensus statement (Kirkpatrick et al., 2006) there are five specific sub-types of negative symptoms: blunted affect, alogia, anhedonia, asociality, and avolition. These can be grouped into two major domains: expressive deficits (blunted affect, alogia) and avolition (anhedonia, asociality, avolition). We created a score for each symptom sub-type by summing individual questions, although alogia, anhedonia and asociality were measure by single questions (eFig. 1). We also created two major domain scores; total score for expressive deficits ranged from 0 to 3, and that for avolition ranged from 0 to 7.

### 2.5 Assessment of potential confounders

Ethnicity and father's occupation were recorded during pregnancy; sex was recorded at birth; body mass index (BMI) was recorded around blood collection for CRP assay; age (in months) was recorded at the time of assessment of psychotic symptoms. Father's occupation was coded in six categories as per the UK Office of National Statistics classification system: I, II, III non-manual, III manual, IV, V (in descending order with professionals and higher managerial workers representing social class I). Depressive symptoms were assessed at the time of assessment of psychotic symptoms using the validated short mood and feelings questionnaire (SMFQ) (Angold et al., 1995). The SMFQ comprises 13 items covering core symptoms of depression and anxiety experienced in past two weeks. Each item is scored zero (not true), one (sometimes true) or two (true) giving a total score of 0–26.

Smoking, alcohol, cannabis and other drug use were recorded using a questionnaire administered alongside assessment of psychotic symptoms. Number of cigarettes/roll ups smoked per day was coded as categorical variable: do not smoke; 1–5; 6–10; >10 cigarettes. Frequency of alcohol use was coded as never; monthly or less frequently; two to four times a month; twice a week or more frequently. The amount of cannabis used in past three months was coded as nil; <8th of an ounce; >8th of an ounce. Use of any other drugs since 15th birthday was coded as a single binary variable. Other drugs included inhaling/sniffing gas, solvent, aerosol, glue/poppers (alkyl nitrites); stimulants (amphetamine, MDMA, cocaine/crack cocaine), hallucinogens (LSD, magic mushroom); heroin, ketamine, anabolic steroids.

### 2.6 Statistical analysis

#### 2.6.1 Baseline comparisons

Distributions of covariates were compared among groups with low, medium and high levels of CRP at baseline using one-way analysis of variance for continuous variables (e.g., age) and Chi-squared test for categorical variables (e.g., sex). We tested correlation of total negative symptom score with any positive symptoms (binary) using point bi-serial correlation, and with total SMFQ score using Spearman's correlation.

#### 2.6.2 Association of CRP with positive and negative symptom dimension scores

First, we carried out exploratory factor analysis (EFA) based on 10 positive and 10 negative symptoms to test whether these symptoms represent one, two or three underlying factors. The two-factor solution representing positive and negative symptoms was chosen as optimum based on the interpretability of factors, and model fit indices (comparative fit index (CFI), Tucker-Lewis index (TLI), and Root Mean Square Error of Approximation (RMSEA)) obtained using MPlus software (Muthén and Muthén, 1998–2011). Then, we extended the factor analytic model to test association between CRP and positive and negative symptom factors scores.

#### 2.6.3 Association between CRP and individual positive symptoms

We used logistic regression to calculate odds ratio (OR) and 95% confidence intervals (CI) for common, individual symptoms (auditory hallucinations, visual hallucinations and paranoid belief) for participants with medium and high, compared with low, CRP levels. ORs were also calculated for any hallucinations, delusions and thought interference (separately). All regression models were adjusted for age, sex, BMI, father's occupation, ethnicity, total SMFQ score, smoking, alcohol, cannabis and other drug use.

#### 2.6.4 Association between CRP and individual negative symptoms

We used logistic regression to examine association between CRP (continuous variable; Z-transformed) and anhedonia, asociality and alogia, so the ORs represent increase in risk of each symptom per SD increase in CRP. We used linear regression for the other negative symptom subtypes and two major domain scores as these were coded as continuous variables. Regression models were adjusted for all confounders mentioned above.

#### 2.6.5 Sensitivity analyses

We re-examined the association between CRP and auditory hallucinations after excluding participants who had reported to experience any positive symptoms only in the context of substance use (within 24 h of using cannabis or other drugs), illness (fever) or sleep (falling asleep/waking up). We also re-examined this association after excluding participants who had previously been assessed to have any positive symptoms at age 12 years (Zammit et al., 2008) to minimize reverse causality. We also reassessed the main findings after excluding participants with CRP>10 mg/L from the sample as this may indicate current infection.

#### 2.6.6 Correction for multiple testing

*P*-values for unadjusted ORs for specific positive/negative symptoms were corrected using Holm-Bonferroni method (Holm, 1979).

## 3 Results

### 3.1 Baseline characteristics of sample

Around age 16 out of all 3488 participants with blood data, 263 (7.5%) had high CRP levels (>3 mg/L). CRP associated with BMI and tobacco, cannabis and other drug use, but not with sociodemographic factors or total SMFQ score (Table 1).

**Table 1:** Baseline characteristics of sample.

Characteristic	Serum CRP level at age 16 years			*P-*Value^3^
	Low (>1 mg/L)	Medium (1–3 mg/L)	High (>3 mg/L)	
Total Sample, No. (%)	2685 (77.0)	540 (15.5)	263 (7.5)	–
Female Sex, No. (%)	1371 (51.1)	285 (52.8)	133 (50.6)	0.74
British White Ethnicity, No. (%)	2423 (98.2)	475 (97.3)	229 (97.4)	0.45
Father's Occupation, No. (%)				0.17
I	342 (15.0)	59 (13.1)	17 (7.8)	
II	860 (37.7)	161 (35.9)	78 (35.9)	
III non-manual	282 (12.4)	51 (11.4)	26 (12.0)	
III manual	581 (25.5)	127 (28.3)	70 (32.3)	
IV	171 (7.5)	41 (9.1)	20 (9.2)	
V	41 (1.8)	10 (2.2)	6 (2.8)	
BMI around CRP assessment, Mean (SD)^a^	21.00 (2.94)	23.36 (4.54)	23.40 (4.89)	<0.001
Age at Assessment of Psychotic Symptoms, mean (SD), Months	200 (2.83)	200 (2.89)	200 (2.73)	0.89
Total SMFQ^b^ Score at Assessment of Psychotic Symptoms, Mean (SD)	5.67 (5.41)	5.64 (5.59)	6.24 (6.13)	0.44
Cigarettes smoked per day, No. (%)				0.07
Do not smoke	1685 (88.1)	310 (88.8)	135 (85.4)	
1-5 cigarettes	114 (6.0)	13 (3.7)	8 (5.1)	
6-10 cigarettes	75 (3.9)	13 (3.7)	7 (4.4)	
>10 cigarettes	38 (2.0)	13 (3.7)	8 (5.1)	
Frequency of alcohol use, No. (%)				0.66
Never, monthly or less frequently	821 (42.9)	158 45.3)	66 (41.8)	
Two to four times a month	790 (41.3)	132 (37.8)	70 (44.3)	
Twice a week or more frequently	301 (15.7)	59 (16.9)	22 (13.9)	
Amount of cannabis used in past three months, No (%)				0.01
Nil	1626 (85.0)	298 (85.5)	128 (81.0)	
<8th of an ounce	215 (11.2)	44 (12.6)	17 (10.8)	
>8th of an ounce	71 (3.7)	7 (2.0)	13 (8.2)	
Use of any other drug since 15th birthday^3^, No. (%)	286 (15.7)	47 (14.5)	34 (23.8)	0.02

^3^ Other drugs included inhaling/sniffing of gas, solvent, aerosol, glue or poppers; use of stimulants (amphetamine, MDMA, cocaine or crack cocaine), hallucinogens (LSD, magic mushroom); heroin, ketamine, and anabolic steroids.

^3^ For continuous variables One Way Analysis of Variance was used to compare mean values among groups (age and total SMFQ score at assessment of psychotic symptoms); For categorical variables Chi-squared test was used to compare proportions among groups (sex, ethnicity, father's occupation, smoking, cannabis, alcohol, and other drug use).

a BMI=Body Mass Index.

b SMFQ=Short Mood and Feelings Questionnaire.

### 3.2 Prevalence of positive symptoms of psychosis

**Fig. 1:**
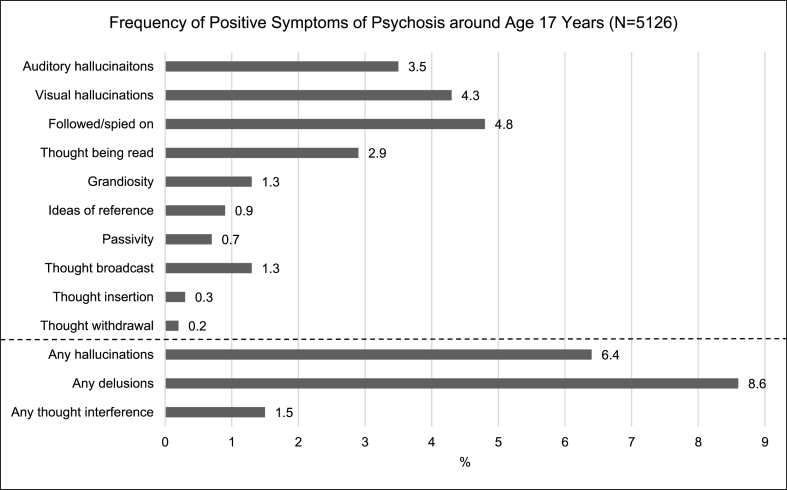
Frequency of positive symptoms of psychosis around age 17 Years in the alspac birth cohort.

Around age 17 out of total 5126 participants assessed, 682 (13.3%) reported at least one positive symptom (eTable 3). Paranoid beliefs of being followed or spied on (4.8%), visual hallucinations (4.3%) and auditory hallucinations (3.5%) were most common (Fig. 1).

### 3.3 Relationship of negative symptoms with positive and mood symptoms

The median (IQR) for total negative symptom score was 0 (0–2). Total negative symptom score was more strongly correlated with total SMFQ score (Spearman's rho*=*0.51; *P* < 0.001; N = 4981) than positive symptoms (*r*_pb_*=*0.19; *P* < 0.001; N = 5108).

### 3.4 Association of CRP with positive and negative symptom dimension scores

**Fig. 2:**
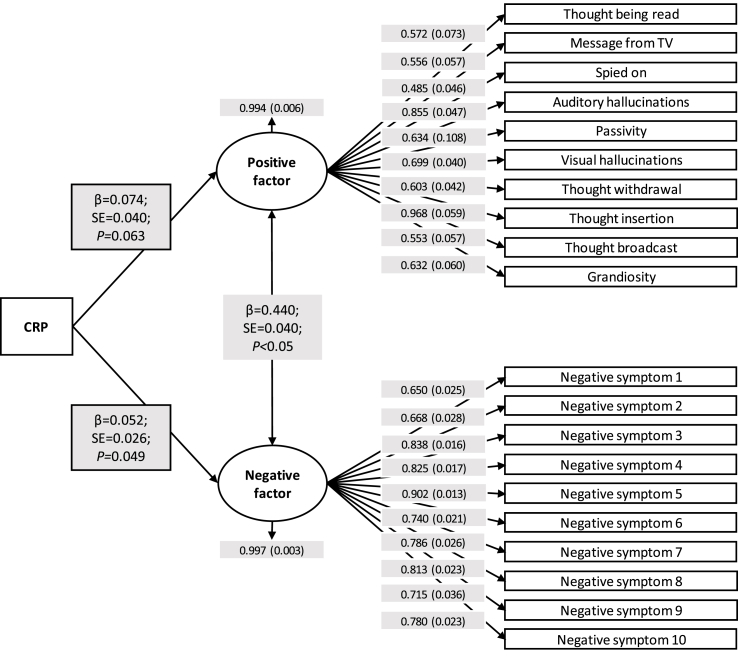
Structural equation model for association between CRP and positive and negative symptom dimensions.

EFA of 10 positive and 10 negative symptoms showed that the two-factor model was optimum based on model fit indices, parsimony and interpretability (eTable 4). Two factors represented positive and negative symptom dimensions, which were correlated; *r=*0.44 (*P* < 0.05). The association of CRP with positive (β = 0.074; SE = 0.040; *P* = 0.063) and negative (β = 0.052; SE = 0.026; *P* = 0.049) symptom dimension scores was similar (Fig. 2).

### 3.5 Association between CRP and specific positive symptoms

High, compared with low, serum CRP levels were associated with auditory hallucinations after controlling for age, sex, BMI, father's occupation, ethnicity, depressive symptoms, smoking, alcohol, cannabis and other drug use; adjusted OR = 2.22 (95% CI, 1.04–4.76) (Table 2). CRP was not associated with any other positive symptoms.

**Table 2:** Odds ratio for positive symptoms of psychosis around age 17 Years for serum CRP levels around age 16 Years in the ALSPAC birth cohort.

Outcome	CRP Level	Sample	Symptom Present, No. (%)	Odds Ratio (95% CI) for Positive Symptoms		
				Unadjusted	Adjusted for age at outcome, sex, BMI, father's occupation, ethnicity, and total SMFQ score^a^	Additional adjustment for smoking, alcohol, cannabis and other drug use^b^
**Auditory Hallucination**						
	Low (<1 mg/L)	1912	63 (3.3)	1 [Reference]	1 [Reference]	1 [Reference]
	Medium (1–3 mg/L)	351	19 (5.4)	1.68 (1.00–2.84)	1.51 (0.81–2.81)	1.23 (0.62–2.44)
	High (>3 mg/L)	158	12 (7.6)	2.41 (1.27–4.57)	2.14 (1.03–4.47)	2.22 (1.04–4.76)
**Visual Hallucination**						
	Low (<1 mg/L)	1912	78 (4.1)	1 [Reference]	1 [Reference]	1 [Reference]
	Medium (1–3 mg/L)	351	13 (3.7)	0.90 (0.50–1.64)	0.65 (0.32–1.36)	0.66 (0.31–1.42)
	High (>3 mg/L)	158	11 (7.0)	1.76 (0.92–3.38)	1.44 (0.67–3.10)	0.92 (0.36–2.33)
**Paranoid Beliefs of Being Followed/Spied On**						
	Low (<1 mg/L)	1912	83 (4.3)	1 [Reference]	1 [Reference]	1 [Reference]
	Medium (1–3 mg/L)	351	17 (4.8)	1.12 (0.65–1.91)	1.22 (0.66–2.24)	1.32 (0.71–2.84)
	High (>3 mg/L)	158	12 (7.6)	1.81 (0.97–3.40)	2.07 (1.03–4.19)	1.85 (0.85–4.01)
**Any Hallucinations**						
	Low (<1 mg/L)	1912	116 (6.1)	1 [Reference]	1 [Reference]	1 [Reference]
	Medium (1–3 mg/L)	351	24 (6.8)	1.13 (0.72–1.79)	0.96 (0.56–1.65)	0.84 (0.46–1.52)
	High (>3 mg/L)	158	18 (11.4)	2.00 (1.17–3.36)	1.78 (0.97–3.27)	1.43 (0.73–2.82)
**Any Delusions**						
	Low (<1 mg/L)	1912	162 (8.5)	1 [Reference]	1 [Reference]	1 [Reference]
	Medium (1–3 mg/L)	351	25 (7.1)	0.82 (0.53–1.28)	0.82 (0.49–1.37)	0.87 (0.52–1.46)
	High (>3 mg/L)	158	20 (12.7)	1.56 (0.95–2.57)	1.37 (0.75–2.51)	1.30 (0.70–2.46)
**Any Thought Interferences**						
	Low (<1 mg/L)	1912	27 (1.4)	1 [Reference]	1 [Reference]	1 [Reference]
	Medium (1–3 mg/L)	351	4 (1.1)	0.80 (0.28–2.31)	0.95 (0.31–2.99)	0.76 (0.21–2.84)
	High (>3 mg/L)	158	1 (0.6)	0.44 (0.06–3.29)	0.52 (0.06–4.02)	0.48 (0.06–3.80)

a Short Mood and Feelings Questionnaire (SMFQ) was completed by participants at the time of questionnaire assessment of psychotic symptoms. BMI was assessed around blood collection for CRP assay.

b Number of cigarettes smoked per day, frequency of alcohol use, amount of cannabis used in past three months, and use of other drugs since 15th birthday were recorded using a questionnaire at the time of assessment of psychotic symptoms. Other drugs included inhaling/sniffing of gas, solvent, aerosol, glue or poppers (alkyl nitrites); use of stimulants (amphetamine, MDMA, cocaine or crack cocaine), hallucinogens (LSD, magic mushroom); heroin, ketamine, and anabolic steroids.

### 3.6 Association between CRP and specific negative symptoms

CRP was associated with anhedonia. The OR for anhedonia per SD increase in serum CRP levels was 1.13 (95% CI, 1.04–1.23); *P=*0.003. Evidence for this association remained after adjusting for current mood (*P=*0.007), and all potential confounders (*P=*0.023) (Table 3). The association of CRP with asociality and alogia was no longer significant after adjusting for confounders. Out of two negative symptom domains, after adjusting for potential confounders CRP was associated with avolition (which includes anhedonia) (regression co-efficient = 0.057; SE = 0.028; *P* = 0.042), but not with expressive deficit (eTable 5).

**Table 3:** Association between serum CRP level around age 16 Years and negative symptoms of psychosis around age 17 Years in the ALSPAC birth cohort.

Symptom Subtype	Adjustment for Confounders	Sample	OR (95% CI)^d^	*P-*Value
**Anhedonia**	Unadjusted analysis	2419	1.13 (1.04–1.23)	0.003
	Adjusted for age at outcome, sex, BMI, father's occupation, and ethnicity	2078	1.15 (1.05–1.26)	0.001
	Adjusted for total SMFQ score^a^	2371	1.14 (1.03–1.25)	0.007
	Adjusted for substance misuse^b^	2293	1.11 (1.02–1.22)	0.022
	Fully adjusted^c^	1944	1.13 (1.02–1.26)	0.023
**Asociality**	Unadjusted analysis	2419	1.13 (1.02–1.25)	0.020
	Adjusted for age at outcome, sex, BMI, father's occupation, and ethnicity	2078	1.13 (1.02–1.26)	0.022
	Adjusted for total SMFQ score^a^	2371	1.11 (0.98–1.26)	0.084
	Adjusted for substance misuse^b^	2293	1.16 (1.04–1.29)	0.008
	Fully adjusted^c^	1944	1.14 (1.00–1.31)	0.064
**Alogia**	Unadjusted analysis	2419	1.08 (1.00–1.18)	0.067
	Adjusted for age at outcome, sex, BMI, father's occupation, and ethnicity	2078	1.09 (1.01–1.19)	0.036
	Adjusted for total SMFQ score^a^	2371	1.07 (0.97–1.17)	0.169
	Adjusted for substance misuse^b^	2293	1.06 (0.96–1.17)	0.236
	Fully adjusted^c^	1944	1.05 (0.94–1.17)	0.365
**Symptom Subtype**	**Adjustment for Confounders**	**Sample**	**Regression Co-efficient (SE)**^e^	***P-*Value**
**Avolition**	Unadjusted analysis	2419	0.040 (0.023)	0.087
	Adjusted for age at outcome, sex, BMI, father's occupation, and ethnicity	2078	0.047 (0.23)	0.047
	Adjusted for total SMFQ score^a^	2371	0.024 (0.020)	0.229
	Adjusted for substance misuse^b^	2293	0.038 (0.025)	0.122
	Fully adjusted^c^	1944	0.027 (0.022)	0.209
**Blunted Affect**	Unadjusted analysis	2419	0.004 (0.009)	0.693
	Adjusted for age at outcome, sex, BMI, father's occupation, and ethnicity	2078	0.002 (0.009)	0.820
	Adjusted for total SMFQ score^a^	2371	−0.002 (0.008)	0.768
	Adjusted for substance misuse^b^	2293	−0.003 (0.010)	0.797
	Fully adjusted^c^	1944	−0.009 (0.009)	0.310

a Short Mood and Feelings Questionnaire (SMFQ) was completed by participants at the time of questionnaire assessment of negative symptoms.

b Number of cigarettes smoked per day, frequency of alcohol use, amount of cannabis used in past three months, and use of other drugs since 15th birthday were recorded using a questionnaire at the time of assessment of psychotic symptoms. Other drugs included inhaling/sniffing of gas, solvent, aerosol, glue or poppers (alkyl nitrites); use of stimulants (amphetamine, MDMA, cocaine or crack cocaine), hallucinogens (LSD, magic mushroom); heroin, ketamine, and anabolic steroids.

c Adjusted for all co-variates, i.e. age at outcome, sex, BMI, father's occupation, ethnicity, total SMFQ score, smoking, alcohol, cannabis and other drug use.

d Logistic regression was used to calculate OR and 95% confidence intervals for each symptom per SD increase in serum CRP level.

e Linear regression was used to calculate regression coefficient and standard error for each symptom per SD increase in serum CRP level.

### 3.7 Results for sensitivity analyses

Evidence for association between CRP levels and auditory hallucinations remained after excluding participants who experienced positive symptoms in the context of cannabis/other drug use, illness or sleep; adjusted OR = 2.49 (95% CI, 1.01–6.17). Evidence for this association remained after excluding participants who had reported any psychotic symptom previously at age 12 years; adjusted OR = 4.52 (95% CI, 1.84–11.08). Evidence for association between CRP and auditory hallucinations also remained after excluding participants with CRP>10 mg/L at baseline; adjusted OR = 2.82 (95% CI, 1.25–6.37); please see eTable 6. After excluding participants with CRP>10 mg/L at baseline, the adjusted OR for anhedonia per SD increase in CRP remained similar to the primary results, but the 95% CI widened and included the null; adjusted OR = 1.21 (95% CI, 0.80–1.84).

### 3.8 Correction for multiple testing

After Holm-Bonferroni correction, the *P-*values for unadjusted ORs for auditory hallucinations (*P=*0.042) and anhedonia (*P=*0.015) remained significant (eTable 7-8).

## 4 Discussion

Based on a general population-based cohort of adolescents, our findings indicate that inflammation is associated with auditory hallucinations and anhedonia specifically out of all twenty positive and negative symptoms studied. Evidence for these associations persisted after adjustments for sex, body mass index, depressive symptoms, sociodemographic factors and substance use, and after correction for multiple testing. Evidence for association between CRP and auditory hallucinations also remained in sensitivity analyses focusing on more clinically relevant symptoms or excluding participants with preexisting symptoms. Negative symptoms were strongly correlated with depressive symptoms, but evidence for association between CRP and anhedonia remained after adjusting for depressive symptoms.

Psychotic symptoms reported by adolescents/adults in general population exist on a continuum with that in schizophrenia (van Os et al., 2009). Longitudinal studies suggest that psychotic symptoms in childhood/adolescence are associated with increased risks for psychosis (Poulton et al., 2000; Zammit et al., 2013) and depression (Rossler et al., 2011) subsequently in adulthood. Therefore, these symptoms are considered as important risk markers for a wide range of psychiatric disorders (Kelleher et al., 2012b). Previous studies have reported an association of CRP with negative symptoms (Boozalis et al., 2017), general psychopathology (Fan et al., 2007) and cognitive dysfunction (Dickerson et al., 2007; Johnsen et al., 2016) in patients with psychosis. Our findings suggest that inflammation is also associated with sub-clinical manifestations of psychosis in general population.

Association of inflammation with symptoms that commonly occur in different psychiatric disorders may be one explanation for the reported trans-diagnostic effect of inflammation. Anhedonia and auditory hallucinations occur in a number of psychiatric disorders including schizophrenia and depression (APA, 2013). Inflammation has been implicated in both of these disorders (Dantzer et al., 2008; Khandaker et al., 2015; Miller et al., 2009, 2011). Inflammation is associated with anhedonia like behavior in animals and humans, which could be mediated by its effect on striatal dopamine synthesis/release (Capuron et al., 2012) and reward perception (Eisenberger et al., 2010). In non-human primates, chronic, low-dose peripheral interferon administration reduces striatal dopamine release in association with anhedonia-like behavior (Felger et al., 2013). In healthy volunteers, inflammation induces hedonic alterations (decreased preference for reward and increased avoidance of punishment) (Harrison et al., 2016), which resemble anhedonia. In depressed patients, increased plasma CRP levels are associated with anhedonia and psychomotor retardation (Felger et al., 2016).

Inflammation could also influence reward perception by affecting glutamatergic neurotransmission. Inflammatory cytokines activate indoleamine 2,3 dioxygenase (IDO), an enzyme that breaks down tryptophan along the kynurenine pathway, leading to increased levels of kynurenic acid and quinolinic acid (Haroon et al., 2012). Kynurenic acid is the only naturally occurring N-methyl D-Aspartate receptor (NMDAR) antagonist in the human CNS (Schwarcz and Pellicciari, 2002). Concentrations of kynurenic acid are higher in the CSF and brain tissue of schizophrenia patients compared with healthy controls (Schwarcz et al., 2001). Injection of ketamine (an NMDAR antagonist) in healthy volunteers has been reported to produce phenomena resembling negative symptoms (Pomarol-Clotet et al., 2006).

Strengths of the work include use of a longitudinal, general population-based sample, data on positive and negative symptoms, and a number of relevant confounders including depressive symptoms and substance misuse. We used two different analytic approaches, regression and factor analysis, which yielded consistent results. A key limitation of the study is the use of self-reported symptoms, as lack of scrutiny from an interviewer and misinterpretation of questions by participants could contribute to measurement error especially for negative symptoms. To minimize such error, we recoded individual positive and negative symptoms into binary variables to ensure that only symptoms reported as definitely present or often/always present were coded as ‘yes’. The prevalence of these symptoms, such as hallucinations in our sample, were not overly high, suggesting that participants did not endorse symptoms wholesale. Majority of participants had attended a face-to-face interview for psychotic symptoms previously at age 12 with a trained assessor (Zammit et al., 2013), so they would have been familiar with what the questionnaires were trying to establish.

The prevalence of any positive symptom in this adolescent sample (13.3%) is comparable to that in other studies. According to a systematic review of population-based studies, the median prevalence of questionnaire-assessed positive symptoms in childhood/adolescence is around 9% (prevalence range from 4.7 to 35.3%) (Kelleher et al., 2012a). Studies of psychotic symptoms particularly those based on general population samples have often focused only on positive symptoms (Kelleher et al., 2012a), so this is one of the few studies of this kind to include negative symptoms. High prevalence is unlikely to explain the association of auditory hallucinations with CRP. CRP was not associated with visual hallucinations or paranoid ideation, which were more common than auditory hallucinations.

The length of follow-up for this longitudinal study was approximately one year; CRP was measured around age 16 and psychotic symptoms were assessed around age 17. However, the questionnaire elicited occurrence of positive symptoms anytime since 15th birthday, so the possibility that some participants might have experienced these symptoms before CRP measurement cannot be ruled out. It is noteworthy that evidence for association between CRP and auditory hallucinations persisted even after excluding participants who had reported any psychotic symptoms previously at age 12.

Other limitations include use of one off measurement for CRP and lack of data on other inflammatory markers. However, CRP is a reliable marker of low-grade inflammation that has been used extensively in the literature. Anhedonia, asociality and alogia were measured using a single question, so in future studies should use more detailed assessments for these symptoms. Number of cases for some of the rare symptoms, such as thought interference, were relatively small, so in future studies with larger samples are required.

Mechanisms for how inflammation cause auditory hallucinations require further investigation. Childhood abuse/maltreatment is associated with increased risk of auditory hallucinations, psychotic disorders (Sheffield et al., 2013) and with increased concentrations inflammatory markers (Baumeister et al., 2016). Whether low-grade inflammation mediates the relationship between childhood maltreatment and auditory hallucinations in adolescence or adult psychotic disorders is an important hypothesis that future studies should address.

Associations of CRP with positive and negative symptom factor scores were similar. However, composite scores can sometimes mask heterogeneity of association at individual symptom level in the case of heterogeneous syndromes like psychosis or depression, as evident from our data. A symptom-based approach may help to provide a better understanding of the role of inflammation in psychiatric disorders. For instance, our findings suggest that association of CRP with symptoms commonly shared between mood and psychotic disorders, such as auditory hallucinations and anhedonia, could be one explanation for the apparent trans-diagnostic effect of inflammation. Using a symptom-based approach, emerging evidence provides some clarity regarding potential role of inflammation in depression. Findings from the Dutch NESDA cohort (Duivis et al., 2013), UK ALSPAC cohort (Chu et al., 2019), US cohorts (Jokela et al., 2016), and from clinical sample (Kohler-Forsberg et al., 2017) suggest that inflammation is mainly associated with somatic/neurovegetative symptoms (e.g., fatigue, sleep disturbance) rather than psychological symptoms of depression. Such evidence could help to inform new and more personalized approaches to treatment and prevention of psychiatric disorders.

A clearer understanding of inflammation-related phenotypes could inform clinical trials of anti-inflammatory drugs for depression and schizophrenia. Elevated inflammatory markers at baseline predict poor antipsychotic response in first episode psychosis (Mondelli et al., 2015). Anti-inflammatory drugs may be helpful for some, but not all, patients with depression, especially those with evidence of inflammation (Kappelmann et al., 2018; Raison et al., 2013). Therefore, a more personalized/stratified approach to sample selection in RCTs is required. Patients with psychosis who show evidence of inflammation and inflammation related symptoms, such as anhedonia and auditory hallucinations, may be better candidates for RCTs of anti-inflammatory drugs.

In summary, we provide evidence that previously reported association of inflammation with psychotic disorders extend to sub-clinical manifestations of psychotic symptoms in young people. Our findings also suggest that association of CRP with symptoms commonly shared between mood and psychotic disorders, such as auditory hallucinations and anhedonia, could be one explanation for the apparent trans-diagnostic effect of inflammation. Replication of these findings in other samples particularly in patients with psychosis are required.

## Funding

Core support for the ALSPAC birth cohort is provided by the: United Kingdom (UK) Medical Research Council (MRC); Wellcome Trust, UK (Grant ref: 102215/2/13/2); and University of Bristol. Dr Khandaker acknowledges grant support from the: Wellcome Trust, UK (201486/Z/16/Z); MRC, UK (MC_PC_17213); and MQ: Transforming Mental Health (MQDS17/40). Professor Jones acknowledges grant support from the: Wellcome Trust, UK (095844/Z/11/Z & 088869/Z/09/Z); National Institute for Health Research (NIHR), UK (RP-PG-0606-1335, Cambridge Biomedical Research Centre and CLAHRC East of England). Prof Dantzer acknowledges grant support from the: National Institute of Health (NIH), USA (MH104694, NS073939); and Cancer Center Support Grant (P30 CA016672). The funding agencies had no role in the design, conduct, and analysis of this study or writing of this manuscript.

## Contributors

GMK designed the study, carried out data analysis and drafted the manuscript. JS contributed to study design, data analysis and revised the manuscript. SZ, GL, RD, and PBJ contributed to study design and revised the manuscript.

## Declaration of competing interest

The authors have no competing financial interests in relation to the work described. Prof Jones received a honorarium that he donated to his department, from Roche (UK), for taking part in an advisory board to advise on education about schizophrenia for psychiatrists. Prof Dantzer has received honorarium from Danone Nutricia Research unrelated to this study.
