# Supplementary material for: Association between circulating levels of C-reactive protein and positive and negative symptoms of psychosis in adolescents in a general population birth cohort
Source: J Psychiatr Res. 2021 Nov;143:534–42. doi: 10.1016/j.jpsychires.2020.11.028 (PMC8126639; doi:10.1016/j.jpsychires.2020.11.028)
Supplement: Multimedia component 1 [file mmc1.docx]

**Online Supplementary Material**

**Khandaker *et al.* Association between Circulating Levels of C-reactive Protein and Positive and Negative Symptoms of Psychosis in Adolescents in a General Population Birth Cohort**

**eFigure 1: Negative Symptom Subtypes and Domains around Age 17 Years based on Self-Reported Questionnaire Data from the ALSPAC Birth Cohort**

**eTable 1: Questions Eliciting Positive Symptoms of Psychosis around Age 17 Years (Sample=5126)**

| Q1 | Some people believe that other people can read their thoughts. Have other people ever read your thoughts? |
| --- | --- |
| Q2 | Have you ever believed that you were being sent special messages through the television or the radio, or that a programme had been arranged just for you alone? |
| Q3 | Have you ever thought you were being followed or spied on? |
| Q4 | Have you ever heard voices that other people couldn't hear? |
| Q5 | Have you ever felt that you were under the control of some special power? |
| Q6 | Have you ever seen something or someone that other people could not see? |
| Q7 | Have you ever felt that your thoughts were being taken out of your head against your will? |
| Q8 | Have you ever felt that someone else's thoughts were being inserted into your head against your will? |
| Q9 | Have you ever felt that your thoughts were so loud that people around you could hear what you were thinking? |
| Q10 | Have you ever felt that you are somebody really very special, or that you have special powers like reading people's minds, or that you have been chosen to perform great and special tasks? (This doesn't mean that you are just clever or that you come from an important family). |

**eTable 2: Questions Eliciting Negative Symptoms around Age 17 Years (Sample=5122)**

| **Question No.** | **Question** | **Response Frequency, No. (%)** | | | |
| --- | --- | --- | --- | --- | --- |
|  |  | **Never** | **Some-times** | **Often** | **Always** |
| Q1 | Have you felt that you are not much of a talker when you are chatting with other people? | 2570 (50.2) | 1696 (33.1) | 635 (12.4) | 221 (4.3) |
| Q2 | Have you felt that you experience few or no emotions at important events, such as on your birthday? | 3486 (68.1) | 1090 (21.3) | 384 (7.5) | 162 (3.2) |
| Q3 | Have you felt that you are lacking in motivation when you have to do things? | 1815 (35.4) | 2325 (45.4) | 735 (14.3) | 247 (4.8) |
| Q4 | Have you felt that you are spending all your days doing nothing? | 2443 (47.7) | 1830 (35.7) | 606 (11.8) | 243 (4.7) |
| Q5 | Have you felt that you are lacking 'get up and go'? | 2071 (40.4) | 2128 (41.5) | 665 (13.0) | 258 (5.0) |
| Q6 | Have you felt that you have only a few hobbies or interests? | 2477 (48.4) | 1634 (31.9) | 674 (13.2) | 336 (6.6) |
| Q7 | Have you felt that you have no interest to be with other people? | 3818 (74.5) | 998 (19.5) | 235 (4.6) | 71 (1.4) |
| Q8 | Have you felt that you are not a very lively person? | 3377 (65.9) | 1361 (26.6) | 281 (5.5) | 103 (2.0) |
| Q9 | Have you felt that you are neglecting your appearance or personal hygiene? | 4177 (81.6) | 778 (15.2) | 127 (2.5) | 40 (0.8) |
| Q10 | Have you felt that you can never get things done? | 2911 (56.8) | 1708 (33.3) | 378 (7.4) | 125 (2.4) |

Note: We recoded each question into a binary variable by coding ‘often’ and ‘always’ as 1=symptom present; ‘never’ and ‘sometimes’ as 0=symptom absent.

**eTable 3: Prevalence of Self-Reported Positive Psychotic Symptoms around Age 17 Years in the ALSPAC Birth Cohort**

| **Symptoms Reported to Have Occurred Definitely At Least Once Since 15^th^ Birthday** | **Sample** | **Symptom Present, No. (%)^1^** |
| --- | --- | --- |
| Auditory Hallucination (heard voices other people couldn't hear) | 5126 | 181 (3.5) |
| Visual Hallucination (seen something/someone other people could not see) | 5126 | 222 (4.3) |
| *Any Hallucinations* | *5126* | *327 (6.4)* |
| Paranoid beliefs (believes to have been followed or spied on) | 5126 | 245 (4.8) |
| Believes someone else has read their thoughts | 5126 | 147 (2.9) |
| Grandiosity (felt they were someone really special or had special powers) | 5126 | 69 (1.3) |
| Ideas of Reference (believes to have received messages from television/radio) | 5126 | 45 (0.9) |
| Passivity (felt they were under the control of a special power) | 5126 | 38 (0.7) |
| *Any Delusions* | *5126* | *443 (8.6)* |
| Thought Broadcast (felt their thoughts were so loud people could hear what they were thinking) | 5126 | 65 (1.3) |
| Thought Insertion (felt someone else's thoughts were inserted into their head against their will) | 5126 | 15 (0.3) |
| Thought Withdrawal (felt their thoughts were being taken out of their head against their will) | 5126 | 12 (0.2) |
| *Any Thought Interferences* | *5126* | *78 (1.5)* |
| *Any Positive Psychotic Symptom* | *5126* | *682 (13.3)* |

^1^ Total number of subjects with a particular subgroup of symptoms (e.g. hallucinations) is smaller than the sum of each symptom within that subgroup because some participants reported to have experienced more than one symptom.

**eTable 4: Goodness of Fit Indices and Factor Loadings for One, Two and Three-factor Models from Exploratory Factor Analysis of Positive and Negative Symptoms (N=5140)**

| **Goodness of Fit Indices^1.2^** | | | | | | |
| --- | --- | --- | --- | --- | --- | --- |
|  | **One-Factor Model** | **Two-factor Model** | | **Three-factor Model** | | |
| CFI | 0.912 | 0.970 | | 0.990 | | |
| TLI | 0.902 | 0.962 | | 0.986 | | |
| RMSEA | 0.040 | 0.025 | | 0.015 | | |
| **Factor Loadings** | | | | | | |
| **Item/Symptom** | **One-Factor Model** | **Two-factor Model** | | **Three-factor Model** | | |
|  | **Factor 1** | **Factor 1** | **Factor 2** | **Factor 1** | **Factor 2** | **Factor 3** |
| Thought insertion | **0.359*** | **0.595*** | -0.013 | **0.600*** | -0.022 | 0.158* |
| Ideas of reference | **0.529*** | **0.775*** | -0.027 | **0.782*** | -0.014 | 0.015 |
| Spied on | **0.393*** | **0.601*** | 0.035 | **0.602*** | 0.023 | 0.204* |
| Auditory hallucination | **0.515*** | **0.764*** | 0.027 | **0.767*** | 0.024 | 0.156* |
| Passivity | **0.485*** | **0.745*** | -0.071 | **0.731*** | -0.035 | -0.137 |
| Visual hallucination | **0.496*** | **0.783*** | 0.003 | **0.789*** | -0.012 | 0.241* |
| Thought withdrawal | **0.866*** | **0.960*** | 0.029 | **0.843*** | 0.141 | **-0.354*** |
| Thought insertion | **0.699*** | **0.903*** | -0.130 | **0.764*** | -0.011 | **-0.463*** |
| Thought broadcasting | **0.534*** | **0.610*** | 0.149* | **0.619*** | 0.158* | 0.014 |
| Grandiosity | **0.476*** | **0.698*** | 0.003 | **0.702*** | 0.018 | -0.024 |
| Negative symptom 1 | **0.608*** | -0.039 | **0.642*** | -0.028 | **0.679*** | **-0.311*** |
| Negative symptom 2 | **0.673*** | 0.153* | **0.608** | 0.172* | **0.617*** | -0.047 |
| Negative symptom 3 | **0.843*** | 0.014 | **0.845*** | 0.056 | **0.787*** | 0.275* |
| Negative symptom 4 | **0.830*** | -0.059 | **0.868*** | -0.024 | **0.827*** | 0.204* |
| Negative symptom 5 | **0.894*** | -0.046 | **0.923*** | -0.018 | **0.861*** | **0.407*** |
| Negative symptom 6 | **0.740*** | 0.023 | **0.740*** | 0.046 | **0.721*** | 0.132 |
| Negative symptom 7 | **0.755*** | 0.030 | **0.753*** | 0.063 | **0.764*** | -0.238* |
| Negative symptom 8 | **0.795*** | -0.015 | **0.816*** | -0.005 | **0.858*** | **-0.328*** |
| Negative symptom 9 | **0.698*** | 0.228* | **0.595*** | 0.244* | **0.600*** | -0.020 |
| Negative symptom 10 | **0.812*** | 0.168* | **0.742*** | 0.194* | **0.727*** | 0.127 |

^1^ CFI=comparative fit index; TLI=Tucker-Lewis index; RMSEA=Root Mean Square Error of Approximation

^2^ The two-factor model provided better fit for the data compared to one-factor solution (χ^2^=636.4; df=19; *P*<0.001). The two factors, corresponding to positive and negative symptoms, were easy to interpret. Although statistically the three-factor model provided better fit for the data compared with two-factor model (χ^2^ =283.1; df=18; *P*<0.001), the third factor was hard to interpret, so this model was rejected.

* Significant at 5% level; values greater than 0.3 are shown in bold.

**eTable 5: Association between Serum CRP Level around Age 16 Years and Two Major Domains of Negative Symptoms around Age 17 Years in the ALSPAC Birth Cohort**

| **Symptom Domain** | **Adjustment for Confounders** | **Sample** | **Regression coefficient (SE)** | ***P-*Value** |
| --- | --- | --- | --- | --- |
| **Avolition** | Unadjusted analysis | 2419 | 0.077 (0.030) | 0.010 |
|  | Adjusted for age at outcome, sex, BMI, father’s occupation, and ethnicity | 2078 | 0.085 (0.030) | 0.005 |
|  | Adjusted for total SMFQ score^1^ | 2371 | 0.056 (0.026) | 0.028 |
|  | Adjusted for substance misuse^2^ | 2293 | 0.072 (0.032) | 0.025 |
|  | Fully adjusted^3^ | 1944 | 0.057 (0.028) | 0.042 |
| **Expressive Deficit** |  |  |  |  |
|  | Unadjusted analysis | 2419 | 0.018 (0.014) | 0.191 |
|  | Adjusted for age at outcome, sex, BMI, father’s occupation, and ethnicity | 2078 | 0.019 (0.014) | 0.184 |
|  | Adjusted for total SMFQ score^1^ | 2371 | 0.009 (0.013) | 0.492 |
|  | Adjusted for substance misuse^2^ | 2293 | 0.007 (0.015) | 0.628 |
|  | Fully adjusted^3^ | 1944 | -0.001 (0.014) | 0.947 |

^1^ Short Mood and Feelings Questionnaire (SMFQ) was completed by participants at the time of questionnaire assessment of negative symptoms.

^2^ Number of cigarettes smoked per day, frequency of alcohol use, amount of cannabis used in past three months, and use of other drugs since 15^th^ birthday were recorded using a questionnaire at the time of assessment of psychotic symptoms. Other drugs included inhaling/sniffing of gas, solvent, aerosol, glue or poppers (alkyl nitrites); use of stimulants (amphetamine, MDMA, cocaine or crack cocaine), hallucinogens (LSD, magic mushroom); heroin, ketamine, and anabolic steroids.

^3^ Adjusted for all co-variates, i.e. age at outcome, sex, BMI, father’s social class, ethnicity, total SMFQ score, smoking, alcohol, cannabis and other drug use.

**eTable 6: Odds Ratios for Auditory Hallucinations around Age 17 Years for Serum CRP Levels around Age 16 Years *after Excluding* Participants Who Reported (i) Positive Symptoms in the Context of Cannabis/Other Drug Use, Physical illness or Asleep (i.e., Attributes); (ii) Any Positive Symptoms at Age 12 Years (i.e., Symptom at 12); (iii) CRP>10mg/L at Age 16 Years**

| **Exclusion Criteria** | **CRP Level** | **Sample** | **Symptom Present, No. (%)** | **Odds Ratio (95% CI) for Auditory Hallucinations at 17 Years** | | |
| --- | --- | --- | --- | --- | --- | --- |
|  |  |  |  | **Unadjusted** | **Adjusted for age at outcome, sex, BMI, father’s occupation, ethnicity, and total SMFQ score^1^** | **Additional adjustment for smoking, alcohol, cannabis and other drug use^2^** |
| **(a) Attributes** |  |  |  |  |  |  |
|  | Low (<1mg/L) | 1789 | 41 (2.3) | 1 [Reference] | 1 [Reference] | 1 [Reference] |
|  | Medium (1-3mg/L) | 329 | 13 (4.0) | 1.75 (0.93-3.31) | 1.84 (0.88-3.86) | 1.30 (0.57-2.99) |
|  | High (>3mg/L) | 142 | 8 (5.6) | 2.54 (1.17-5.54) | 2.45 (1.01-5.94) | 2.49 (1.01-6.17) |
| **(b) Symptom at 12^3^** |  |  |  |  |  |  |
|  | Low (<1mg/L) | 1570 | 30 (1.9) | 1 [Reference] | 1 [Reference] | 1 [Reference] |
|  | Medium (1-3mg/L) | 299 | 14 (4.7) | 2.52 (1.32-4.81) | 2.65 (1.30-5.38) | 2.26 (1.05-4.87) |
|  | High (>3mg/L) | 125 | 9 (7.2) | 3.98 (1.84-8.58) | 3.70 (1.56-8.79) | 4.52 (1.84-11.08) |
| **(c) CRP>10mg/L** |  |  |  |  |  |  |
|  | Low (<1mg/L) | 1912 | 63 (3.3) | 1 [Reference] | 1 [Reference] | 1 [Reference] |
|  | Medium (1-3mg/L) | 351 | 19 (5.4) | 1.68 (1.00-2.84) | 1.52 (0.82-2.84) | 1.25 (0.63-2.48) |
|  | High (>3mg/L) | 122 | 11 (9.0) | 2.91 (1.49-5.67) | 2.70 (1.23-5.92) | 2.82 (1.25-6.37) |

^1^ Short Mood and Feelings Questionnaire (SMFQ) was completed by participants at the time of questionnaire assessment of psychotic symptoms. BMI was measured around blood collection for CRP assay.

^2^ Number of cigarettes smoked per day, frequency of alcohol use, amount of cannabis used in past three months, and use of other drugs since 15^th^ birthday were recorded using a questionnaire at the time of assessment of psychotic symptoms. Other drugs included inhaling/sniffing of gas, solvent, aerosol, glue or poppers (alkyl nitrites); use of stimulants (amphetamine, MDMA, cocaine or crack cocaine), hallucinogens (LSD, magic mushroom); heroin, ketamine, and anabolic steroids

^3^ Positive psychotic symptoms (hallucinations, delusions and thought interference) were measured by face-to-face interview at age 12 years, which were coded as absent, suspected symptom, definite symptom. We excluded participants who reported a suspected or definite symptom for analysis presented in this Table.

**eTable 7: Corrected *P*-values for the Association of CRP with Specific Positive Symptoms using the Holm–Bonferroni Method**

| **Symptom** | **Original *P-*value^a^** | **Corrected *P-*value** |
| --- | --- | --- |
| Auditory Hallucination | 0.007 | 0.042 |
| Visual Hallucination | 0.090 | 0.256 |
| Paranoid Beliefs of Being Followed/ Spied On | 0.064 | 0.256 |
| Any Hallucinations | 0.010 | 0.050 |
| Any Delusions | 0.076 | 0.256 |
| Any Thought Interferences | 0.428 | 0.428 |

^a^ The *P*-values correspond to unadjusted ORs presented in Table 2. The ORs represent risk of each symptoms at age 17 for participants with high, compared with low, CRP at age 16.

**eTable 8: Corrected *P*-values for the Association of CRP with Specific Negative Symptoms using the Holm–Bonferroni Method**

| **Symptom** | **Original *P-*value^a^** | **Corrected *P-*value** |
| --- | --- | --- |
| Anhedonia | 0.003 | 0.015 |
| Asociality | 0.020 | 0.080 |
| Alogia | 0.067 | 0.201 |
| Avolition | 0.087 | 0.201 |
| Blunted Affect | 0.693 | 0.693 |

^a^ The *P*-values correspond to unadjusted effect estimates presented in Table 3. The effect estimates presented in Table 3 represent: ORs for anhedonia, asociality and alogia per SD increase in CRP levels; regression co-efficient for avolition and blunted effect per SD increase in CRP levels.
